# Comparison of COVID−19-associated inpatient burden by variant period in hospitalized children age <5 years in the United States

**DOI:** 10.3389/fped.2025.1573933

**Published:** 2025-05-22

**Authors:** Kathleen M. Andersen, Maria D. McColgan, Alejandro Cane, Mary M. Moran, Maya Reimbaeva, Santiago M. C. Lopez

**Affiliations:** ^1^ Medical Evidence Generation, Pfizer Inc., New York, NY, United States; ^2^ US Medical and Scientific Affairs, Pfizer Inc., Collegeville, PA, United States; ^3^ Statistical and Data Science, Pfizer Inc., New York, NY, United States; ^4^ US Medical and Scientific Affairs, Pfizer Inc., New York, NY, United States

**Keywords:** COVID 19, SARS-COV-2 variants, invasive mechanical ventilation, intensive care unit, pediatrics, COVID-19 hospitalizations

## Abstract

**Introduction:**

This study evaluated clinical outcomes in children aged <5 years with COVID−19-associated hospitalizations and assessed whether disease severity differed across periods of SARS-CoV-2 variant predominance in the United States.

**Methods:**

Data from the PINC AI™ Healthcare Database during three distinct periods of SARS-CoV-2 variant predominance [pre-Delta (April 2021–June 2021), Delta (July 2021–December 2021), and Omicron (January 2022–July 2023)] were used. Hospital length of stay (LOS), admission to the intensive care unit (ICU), ICU LOS, usage and duration of oxygen supplementation, usage and duration of invasive mechanical ventilation (IMV), and inpatient death were assessed for each period.

**Results:**

Overall, 10,316 children aged <5 years were hospitalized for COVID-19 over the three periods. Most pediatric COVID-19 hospitalization occurred during the Omicron period. In each time period, children aged <1 year were disproportionately affected. For each outcome of interest, there were no statistically significant differences between variant periods. Similar proportions of hospitalized children required oxygen supplementation (pre-Delta 13%; Delta 16%; Omicron 18%), required IMV (pre-Delta 7%; Delta 8%; Omicron 7%) and experienced in-hospital death (pre-Delta 0.7%; Delta 0.9%; Omicron 0.4%). Duration of hospital LOS, ICU LOS and IMV use were also similar.

**Conclusions:**

Despite perception that the omicron variant is less severe, children aged <5 years have a similar risk of severe COVID-19 as they did in earlier variant eras. These results highlight the need for continued preventative measures against severe COVID-19 in children, including routine immunization for eligible children and pregnant people with an updated COVID-19 vaccine.

## 1 Introduction

Coronavirus disease 2019 (COVID-19), caused by infection with severe acute respiratory syndrome coronavirus 2 (SARS-CoV-2), accounted for over 16.5 million cases under the age of 18 years as of April 26, 2023 (1). As of October 2024, COVID-19 was the third leading infectious disease-related cause of death in children 0–17 years of age in the United States (US) (2). Among children, those aged <5 years have the highest COVID-19 associated hospitalization rate (3–5). As context, between October 2022 and April 2024, COVID-19-associated hospitalization rates in children <6 months of age were higher than any other age group besides those ≥75 years of age (6).

SARS-CoV-2 has evolved over time; in the US, following the original strain, major predominant variants included alpha and beta (pre-Delta phase; prevalent from April 2021 to June 2021), Delta (predominating from July 2021 to December 2021), and Omicron variants (emerging in January 2022) (7, 8). Throughout the pandemic and endemic stages of SARS-CoV-2 circulation, COVID-19 severity of disease fluctuated (9, 10), likely due to a combination of factors, including virulence, increase in immunity from natural infection, use of non-pharmacological interventions such as face masks and social distancing, vaccination and treatment availability, and changes in patterns of testing (11, 12). Previous studies have demonstrated reduced severity of COVID-19 disease burden among patients diagnosed in the omicron era in the outpatient setting (13, 14). However, it is not known whether there have been reductions in inpatient severity. Understanding the clinical severity and disease burden of different SARS-CoV-2 variants in the pediatric population can be important in informing the need for continued surveillance and prevention efforts. This study evaluated clinical outcomes in children <5 years of age with COVID-19-associated hospitalizations during different periods of SARS-CoV-2 variant predominance (pre-Delta, Delta, and Omicron) in the US.

## 2 Materials and methods

### 2.1 Study design and data source

This was a retrospective cohort study that used data from the PINC AI (formerly Premier) Healthcare Database (PHD), a large hospital-based administrative healthcare claims database that encompassed approximately 25% of hospital admissions in all ages in the US (15). PHD includes hospitals from all US Census divisions from each of the four geographic regions (Northeast, Midwest, South and West) with a similar distribution to that of the American Hospital Association (AHA). Approximately 27% of PHD hospitals are in rural areas, and 31.2% were teaching facilities. This database, which has been used previously for other related studies (16–20), included patient demographic information, visit-level information (such as length of hospital stay or in-hospital death), hospital characteristics, medication information, and hospital charges/costs. All the data were de-identified in compliance with the Health Insurance Portability and Accountability Act regulations. This study was deemed exempt from Institutional Review Board review pursuant to the terms of the US Department of Health and Human Service's Policy for Protection of Human Research Subjects as a category 4 exemption (Sterling IRB Protocol #10563, Atlanta, GA).

### 2.2 Patient population and inclusion criteria

Children aged <5 years at the time of admission with a primary or secondary admission diagnosis of COVID-19 (ICD-10-CM U07.1 “COVID-19”) from April 2021 through July 2023 were included. This study considered only a first admission during the study period, as readmissions may have had differential clinical presentation. In order to maintain the study population as patients with community-acquired COVID-19, patients were excluded if: (1) COVID-19 was not present on admission; (2) diagnosed with both COVID-19 and RSV, or COVID-19 and influenza, during the same hospital stay, or (3) patients who were born with COVID-19, defined as patient age of zero years with an ICD-10 CM code indicating live birth (Z38.XX).

### 2.3 Patient characteristics and clinical outcomes

The patient characteristics captured included demographic characteristics (age, sex, race, and ethnicity), details of hospitalization (month of admission, insurance type, hospital urbanicity, diagnosis position, and discharge status) and prevalence of comorbid conditions (diabetes, obesity, hypertension, neurological disease, asthma, Down syndrome/chromosomal abnormality, immunity status-immunocompromised/immunocompetent, inflammatory bowel disease, metabolic disease, sickle cell disease, psychiatric disorders, congenital heart condition, congenital lung condition, autoimmune disease, transplant recipient, and other disabilities). The clinical outcomes analyzed included the hospital length of stay (LOS), admission to the intensive care unit (ICU), ICU LOS, usage and duration of oxygen supplementation, usage and duration of invasive mechanical ventilation (IMV), and inpatient death.

### 2.4 Statistical analysis

Data were stratified into three periods based on the variant predominance: Pre-Delta (April 2021–June 2021), Delta (July 2021–December 2021), and Omicron (January 2022–July 2023). Descriptive statistics were utilized to summarize patient characteristics and outcomes. Categorical variables are presented as frequency and percentage of patients in each category, including those with ‘unknown’ or missing values. Continuous variables were presented as means with standard deviations (SD) and medians with first (Q1) and third (Q3) quartiles. *P* values for 3-way comparisons were calculated using Chi-square tests for proportions and Kruskal–Wallis for medians. All data analyses were performed using the statistical software SAS version 9.4 (SAS Institute; Cary, NC, USA).

## 3 Results

### 3.1 Overall results

A total of 10,316 children aged <5 years were hospitalized for COVID-19 over the three periods of variant predominance. The majority of hospitalizations [7,767 (75%)] were recorded during Omicron predominance (Table 1). When stratified by age groups for the overall patient population as well as for each variant period, most COVID-19 hospitalizations were in children <1 year of age [pre-Delta: 142 (46%); Delta: 1,262 (56%); Omicron: 4,352 (56%); Table 1].

**Table 1:** Hospitalization in children aged <5 years by age and COVID-19 variants.

Age groups	Pre-delta	Delta	Omicron	*P* value*
Hospitalizations, 0–4 years, *N* (%)	308 (3%)	2,241 (22%)	7,767 (75%)	
<1 year	142 (46%)	1,262 (56%)	4,352 (56%)	0.002
1 years	75 (24%)	403 (18%)	1,465 (19%)	0.027
2 years	37 (12%)	244 (11%)	808 (10%)	0.564
3 years	34 (11%)	180 (8%)	621 (8%)	0.157
4 years	20 (7%)	152 (7%)	521 (7%)	0.979

N, total number of children.

* *P* values indicate comparisons between the three groups (Pre-Delta vs. Delta vs. Omicron) using Chi-square tests.

### 3.2 Patient demographics and comorbid conditions

Demographic characteristics and prevalence of comorbid conditions for each period of variant predominance, are shown in Table 2. Throughout the three time periods evaluated, the majority of children were male, admitted to urban hospitals and over two thirds had Medicaid insurance (Table 2). The distribution of patient's race differed with each of the variant periods, with proportionally more children who were Black hospitalized during pre-Delta period (86/308; 28%) than in later time frames (Delta: 424/2,241, 19%; Omicron: 1,116/7,767, 14%) and proportionally more children who were Asian hospitalized during Omicron (294; 4%) than earlier time periods (pre-Delta: 7, 2%; Delta: 40, 2%; Table 2).

**Table 2:** Demographic characteristics and comorbid conditions in children aged <5 years hospitalized with COVID-19 distributed by variants.

Characteristic	Period			
	Pre-delta, *N* = 308	Delta, *N* = 2,241	Omicron, *N* = 7,767	*P* value^a^
Sex, *n* (%)				0.015
Female	126 (41%)	1,024 (46%)	3,291 (42%)	
Male	182 (59%)	1,217 (54%)	4,476 (58%)	
Race/Ethnicity, *n* (%)				
Asian, Hispanic/non-Hispanic	7 (2%)	40 (2%)	294 (4%)	<0.001
Black, Hispanic	4 (1%)	19 (0.8%)	66 (0.8%)	NR
Black, non-Hispanic	86 (28%)	424 (19%)	1,116 (14%)	<0.001
Other, Hispanic	30 (10%)	177 (8%)	818 (11%)	0.001
Other, non-Hispanic	19 (6%)	129 (6%)	436 (6%)	0.896
Unknown	53 (17%)	313 (14%)	1,195 (15%)	0.150
White, Hispanic	39 (13%)	319 (14%)	1,095 (14%)	0.757
White, non-Hispanic	70 (23%)	820 (37%)	2,747 (35%)	<0.001
Insurance type, *n* (%)				
Commercial	63 (21%)	542 (24%)	2,137 (28%)	<0.001
Medicaid	232 (75%)	1,532 (68%)	5,187 (67%)	0.004
Medicare	1 (0.3%)	0 (0%)	6 (0.1%)	NR
Other	11 (4%)	117 (5%)	300 (4%)	0.015
Uninsured	1 (0.3%)	50 (2%)	137 (2%)	NR
Hospital census region, *n* (%)				
Midwest	65 (21%)	442 (20%)	1,277 (16%)	<0.001
Northeast	54 (17%)	321 (14%)	1,334 (17%)	<0.001
South	141 (46%)	1,091 (49%)	3,383 (44%)	<0.001
West	48 (16%)	387 (17%)	1,773 (23%)	<0.001
Hospital urbanicity, *n* (%)				0.015
Rural	8 (3%)	100 (5%)	378 (5%)	
Urban	300 (97%)	2,141 (96%)	7,389 (95%)	
Comorbidities, *n* (%)				
No underlying condition	182 (59%)	1,557 (69%)	5,266 (68%)	<0.001
Diabetes	3 (1%)	12 (0.5%)	47 (0.6%)	NR
Obesity/overweight	4 (1%)	13 (0.6%)	35 (0.5%)	NR
Hypertension	2 (0.6%)	32 (1%)	116 (2%)	NR
Neurological disease	22 (7%)	138 (6%)	565 (7%)	0.190
Asthma/reactive airway	26 (8%)	159 (7%)	605 (8%)	0.482
Chronic liver disease	0 (0%)	0 (0%)	0 (0%)	NR
Down syndrome/chromosomal anomaly	7 (2%)	32 (1%)	128 (2%)	0.501
Immunocompromised	77 (25%)	376 (17%)	1,258 (16%)	<0.001
HIV/AIDS	0 (0%)	0 (0%)	0 (0%)	NR
Solid malignancy	5 (2%)	39 (2%)	150 (2%)	NR
Hematologic malignancy	6 (2%)	21 (0.9%)	97 (1%)	0.233
Bone marrow transplant	0 (0%)	0 (0%)	6 (0.1%)	NR
Organ transplant	3 (1%)	12 (0.5%)	34 (0.4%)	NR
Rheumatologic/other inflammatory condition	29 (9%)	94 (4%)	241 (3%)	<0.001
Primary immunodeficiency	11 (4%)	80 (4%)	283 (4%)	0.985
CKD or ESRD	11 (4%)	65 (3%)	254 (3%)	0.634
Other immune condition	27 (9%)	177 (8%)	543 (7%)	0.199
IBD	0 (0%)	2 (0.1%)	2 (0%)	NR
Metabolic disease	2 (0.6%)	21 (0.9%)	77 (1%)	NR
Sickle cell	7 (2%)	61 (3%)	142 (2%)	0.03
Psychiatric disorders	0 (0%)	6 (0.3%)	9 (0.1%)	NR
Congenital heart condition	3 (1%)	9 (0.4%)	30 (0.4%)	NR
Congenital lung condition	0 (0%)	1 (0%)	36 (0.5%)	NR
Autoimmune disease	3 (1%)	13 (0.6%)	50 (0.6%)	NR
Transplant (Bone marrow/Organ)	3 (1%)	12 (0.5%)	39 (0.5%)	NR
Disability^b^	2 (0.6%)	24 (1%)	68 (0.9%)	NR

AIDS, acquired immunodeficiency syndrome; CKD, chronic kidney disease; ESRD, end-stage renal disease; HIV, human immunodeficiency virus; IBD, inflammatory bowel disease; N, total number of children; n, number of children for each parameter; NR, not reported.

^a^ *P* values were not calculated for groups for which there were ≤5 individuals for any variant period.

^b^ Disability includes neurologic, neurodevelopmental, intellectual, physical, vision or hearing impairment.

Across all variant predominance periods, most children had no underlying medical conditions (pre-Delta: 182, 59%; Delta: 1,557, 69%; Omicron: 5,266, 68%). The most common comorbidities across all periods were immunocompromising conditions (pre-Delta: 77, 25%; Delta: 376, 17%; Omicron: 1,258, 16%). The prevalence of comorbidities was similar for nearly all conditions across each period of variant predominance, except for a higher prevalence of inflammatory conditions during the pre-Delta period (pre-Delta: 29, 9%; Delta: 94, 4%; Omicron: 241, 3%; *P* < 0.001).

### 3.3 Clinical outcomes

Clinical outcomes for children aged <5 years hospitalized with COVID 19 for each period of variant predominance, are shown in Table 3. Overall, for all the measured outcomes, there were no significant differences between periods of variant predominance. The median length of hospitalization was two days for each variant period evaluated (*P* > 0.99). Similar proportions of children required oxygen supplementation (pre-Delta 13%; Delta 16%; Omicron 18%; *P* = 0.06), were admitted to ICU (20% vs. 21% vs. 21%, *P* = 0.77) and received IMV (7% vs. 8% vs. 7%, *P* = 0.35). Days with supplemental oxygen, in the ICU and on IMV were also similar across eras. The proportion of in-hospital death was higher during the Delta period compared with pre-Delta and Omicron periods (pre-Delta: 0.7%; Delta: 0.9%; Omicron: 0.4%); statistical significance could not be estimated due to the low numbers.

**Table 3:** Clinical outcomes in children aged <5 years hospitalized with COVID-19 distributed by variants.

Clinical outcomes	Pre-delta, *N* = 308	Delta, *N* = 2,241	Omicron, *N* = 7,767	*P* value^a^
Hospitalization length of stay (days), Median (IQR)	2.0 (1.0, 3.0)	2.0 (1.0, 4.0)	2.0 (1.0, 3.0)	>0.99
Received supplemental O_2_, *n* (%)	41 (13%)	366 (16%)	1,372 (18%)	0.06
Days with supplemental O_2_, Median (IQR)	2.0 (1.0, 3.0)	2.0 (1.0, 4.0)	2.0 (1.0, 3.0)	
Admitted to ICU, *n* (%)	62 (20%)	461 (21%)	1,644 (21%)	0.77
ICU length of stay (days), Median (IQR)	1.0 (1.0, 3.0)	2.0 (1.0, 5.0)	2.0 (1.0, 4.0)	0.37
IMV, *n* (%)	20 (7%)	181 (8%)	564 (7%)	0.35
Days with IMV, Median (IQR)	2.5 (1.5, 4.5)	3.0 (1.0, 10.0)	2.0 (1.0, 5.0)	
In-hospital death, *n* (%)	2 (0.7%)	19 (0.9%)	27 (0.4%)	NR

ICU, intensive care unit; IMV, invasive mechanical ventilation; IQR, interquartile range; N, total number of children; n, number of children for each parameter; NR, not reported; O_2_, oxygen.

^a^ *P* values indicate comparisons between the three groups (Pre-Delta vs. Delta vs. Omicron) using Chi-square tests for proportions and Kruskal–Wallis for medians. *P* values were not calculated for groups for which there were ≤5 individuals for any variant period.

## 4 Discussion

This study assessed and compared the hospitalizations and clinical outcomes of children aged <5 years diagnosed with COVID-19 in the US using a hospital chargemaster database during three periods of SARS-CoV-2 predominance—pre-Delta, Delta, and Omicron. Importantly, for each variant period, most COVID-19 hospitalizations occurred in children <1 year of age and in children that did not have underlying medical conditions. Despite public perception that the Omicron variant leads to less severe disease, clinical outcomes were similar across each period of variant pre-dominance. However, a higher proportion of deaths were observed during the Delta period.

Consistent with published literature (21, 22), our study found the majority of the hospitalizations in children <5 years of age occurred during the Omicron period, with three times as many hospitalizations during periods of Omicron predominance than pre-Delta and Delta combined. Data from COVID-NET assessing COVID-19 hospitalizations among US children aged 0–11 years reported that hospitalization rates among children aged 0–4 years during the peak of hospitalizations in the Omicron period (week ending 8 January, 2022) were about five times than during the peak of hospitalizations in the Delta period (week ending 11 September, 2021; Delta vs. Omicron, 2.9 vs. 15.6 hospitalizations per 100,000 children) (3). Another study from the US assessed incidence of COVID-19 in a cohort of children aged <5 years from September 1, 2021, to January 31, 2022 reported that the incidence rate of COVID-19 was 6–8 times higher during the Omicron period (2.4–5.6 cases per 1,000 person-days) than the Delta period (1.0–1.5 cases per 1,000 person-days) (23).

As noted in our study for each variant period, most COVID-19 hospitalizations were in children ≤1 year of age, which is consistent with previous reports (9, 24–26). A single-center study in the US comparing the laboratory data and clinical outcomes of the different variants (Alpha, Delta, and Omicron) during January 1, 2021–January 15, 2022 reported a higher number of hospitalizations in children aged <1 year as compared to those aged 1–4 years for each variant (<1 year vs. 1–4 years; Alpha: 38.1% vs. 14.3%; Delta: 25.8% vs. 9.0%; Omicron: 52.5% vs. 10.2%) (9). A multicenter cohort study in Europe assessing COVID-19 among children and adolescents aged <18 years during the pre-Delta period (April 1–24, 2020), reported about one-third of the cases (29.2%, 170/582) were among children aged <1 year (25). Another COVID-NET study comparing hospitalizations during Delta and Omicron periods (June 20, 2020–December 18, 2021) among infants aged <6 months reported that weekly hospitalization rate during the Omicron period was eleven-fold higher [95% confidence interval (CI) 4.3–33.3] compared to the Delta period, and it was also higher than that observed for other pediatric age groups (24). Notably, a study in the US assessing deaths due to COVID-19 between August 1, 2021 and July 31, 2022 reported the highest death rate among those aged <1 year as compared to 1–4 years (<1 year vs. 1–4 years, 4.3 vs. 0.6 deaths per 100,000) (26).

Clinical outcomes indicating severity of disease were consistent, with no significant differences across the three periods of variant predominance in our study. The results of our study are in line with those observed in the previous COVID-NET study that reported no increase in indicators of disease severity (24), and other studies that reported no major differences for hospital length of stay, ICU admission, and patients requiring invasive mechanical ventilation across different periods of variant predominance (3, 9, 24).

There are some limitations to this study. The results of our study might not be applicable to the entire US or to areas that were not included in the data source. PINC data are complete for inpatient encounters, but have incomplete capture of outpatient visits, therefore the total burden of COVID-19 outside the hospital, including post-hospitalization could not be assessed. Information on vaccination and general immunization history for the children in this study population were not captured in the data source.

In the US, COVID-19 vaccines are available for individuals aged ≥6 months (27–30). Infants aged <6 months are not eligible for COVID-19 vaccines (31). However, maternal immunization has shown to provide protection to the fetus and the newborn through infancy (31). Furthermore, infants of mothers who received an additional booster dose for COVID-19 were 56.0% less likely to acquire the infection in the first 6 months as compared to those whose mothers did not receive a booster (32). A COVID-NET study in 2024 showed that rates of COVID-19 associated hospitalization in infants <6 months of age are as high as individuals aged ≥75 years and that the percentage of hospitalized infants whose mothers had been vaccinated against COVID-19 during pregnancy was 18% during October 2022–September 2023 and decreased to <5% during October 2023–April 2024 (6). Our results indicate that infants aged <1 year were disproportionately affected and had a higher risk of SARS-CoV-2 infection. Hence, improved and constant COVID-19 surveillance in the pediatric population along with infection prevention strategies, including routine immunization and vaccination of women who are pregnant, are recommended.

## 5 Conclusion

Data from this large retrospective analysis of COVID19 hospitalizations during the three periods of variant predominance showed that compared with children 1 through 4 years of age, children aged <1 year were disproportionately affected, most hospitalized children had no underlying medical conditions and inpatient COVID-19 severity has not eased with omicron variant predominance. These results highlight the need for continued COVID-19 surveillance in the pediatric population and continued preventative measures against severe COVID-19 in children, including routine immunization for children and pregnant people with an updated COVID-19 vaccine.

## Data Availability

The original contributions presented in the study are included in the article/Supplementary Material, further inquiries can be directed to the corresponding author.
